# Car Seats with Capacitive ECG Electrodes Can Detect Cardiac Pacemaker Spikes

**DOI:** 10.3390/s20216288

**Published:** 2020-11-04

**Authors:** Durmus Umutcan Uguz, Rosalia Dettori, Andreas Napp, Marian Walter, Nikolaus Marx, Steffen Leonhardt, Christoph Hoog Antink

**Affiliations:** 1 Medical Information Technology, Helmholtz-Institute for Biomedical Engineering, RWTH Aachen University, Pauwelsstr. 20, 52074 Aachen, Germany; walter@hia.rwth-aachen.de (M.W.); leonhardt@hia.rwth-aachen.de (S.L.); hoog.antink@hia.rwth-aachen.de (C.H.A.); 2 Department of Cardiology, Pneumology, Angiology and Intensive Care Medicine, University Hospital RWTH Aachen, Pauwelsstr. 30, 52074 Aachen, Germany; rdettori@ukaachen.de (R.D.); anapp@ukaachen.de (A.N.); nmarx@ukaachen.de (N.M.)

**Keywords:** capacitive ECG, cardiac pacemaker, unobtrusive monitoring, noncontact ECG, car seat

## Abstract

The capacitive electrocardiograph (cECG) has been tested for several measurement scenarios, including hospital beds, car seats and chairs since it was first proposed. The inferior signal quality of the cECG compared to the gold standard ECG guides the ongoing research in the direction of out-of-hospital applications, where unobtrusiveness is sought and high-level diagnostic signal quality is not essential. This study aims to expand the application range of cECG not in terms of the measurement scenario but in the profile of the subjects by including subjects with implanted cardiac pacemakers. Within this study, 20 patients with cardiac pacemakers were recruited during their clinical device follow-up and cECG measurements were conducted using a seat equipped with integrated cECG electrodes. The multichannel cECG recordings of active unipolar and bipolar pacemaker stimulation were analyzed offline and evaluated in terms of $F_{\beta }$ scores using a pacemaker spike detection algorithm. $F_{\beta }$ scores from 3652 pacing events, varying from 0.62 to 0.78, are presented with influencing parameters in the algorithm and the comparison of cECG channels. By tuning the parameters of the algorithm, different ranges of $F_{\beta }$ scores were found as 0.32 to 0.49 and 0.78 to 0.88 for bipolar and unipolar stimulations, respectively. For the first time, this study shows the feasibility of a cECG system allowing health monitoring in daily use on subjects wearing cardiac pacemakers.

## 1. Introduction

Cardiovascular diseases (CVD) are responsible for 30% of all deaths and represent the predominant causes of mortality. Among noncommunicable diseases, CVD account for 48% of all deaths and 35% of the causes of death for people younger than 60 years old [1]. Therefore, CVD is not specific to a group of people. On the contrary, it is a public health problem where early diagnosis, prevention and scaled-up monitoring are crucial. Cardiac rhythm abnormalities impair the coordination of the atria and ventricles, which can be treated by implantable cardiac pacemakers. From 2009 to 2013 in Europe, an average of 953 people per million inhabitants per year have received a pacemaker or a cardiac resynchronization device that is essentially a biventricular pacemaker [2]. The doubling of the prevalence rate in a developed economy is faster than 15 years, as opposed to the expected rate in the early 1990s [3]. Briefly, people with implanted cardiac pacemakers already constitute a significant part of society and their number is expected to increase further.

Research on the capacitive electrocardiogram (cECG) has gained momentum in recent years, which promises a way of unobtrusive monitoring of electrical heart activity. The cECG is not considered to be a replacement for the gold standard conductive ECG due to its higher susceptibility to noise and usually lower signal quality. Instead, its applications can be grouped in two fields. In the first scenario, cECG could be used where classical ECG becomes a burden. Lim et al. [4] has suggested patient beds with cECG electrodes. They could relieve the medical staff from replacing electrodes, which would also reduce the negative effects of drying electrodes and skin irritations in long-term monitoring. In the second scenario, cECG can extend ECG monitoring from hospitals to everyday life, which can improve the healthcare systems through prevention and early diagnosis. As an example, the feasibility of car seats for the cECG measurement of cardiac patients was shown in simulated driving [5].

In contrast to classical ECG, where the electrodes need to be attached to the subject’s body firmly, the electrodes in cECG form an electrical contact through the clothing of the subject assumed to form a plate capacitor [6]. The lack of conductive contact translates into a higher coupling impedance [7] that degrades the common-mode rejection ratio and prolongs the dispersion of triboelectric surface charges [8]. Moreover, the susceptibility of the clothing thickness to the changes in the contact pressure can cause additive and multiplicative noise [9] that can even be induced by the mechanical vibrations resulting from the contraction of the heart [10]. Because of these drawbacks, research in cECG is currently concentrating on new applications or technical solutions tested mostly on small groups of subjects without prioritizing the clinical relevance.

Several cECG applications offering unobtrusive monitoring of cardiac activity have already been presented in the literature as an extension of cardiac monitoring to everyday life. However, patients with electrically active implants have been excluded from these studies as a precaution, although these subjects constitute the group that may benefit greatly. They are already diagnosed with CVD and have a higher risk of cardiac events that may occur under the increased risk due to the stress caused while driving [11]. In addition to the risk of an acute event, the prolonged activity of driving provokes a stress response in the autonomous nervous system [12], which may increase the overall risk of these patients. Monitoring of cardiac activity while driving may ensure the well-being of the driver and even enable remote examinations by a cardiologist in necessary cases. Furthermore, cardiac monitoring of the driver may become even more important in the close future. With fully autonomous cars being out of reach for the foreseeable future [13], a semi-automatic driving scenario may utilize cardiac monitoring to ensure the physical state of the driver for switching the control back and forth. For example, the activity of an implanted defibrillator often causes temporary impairment [14] and reduced driving capability, which would be important to know, as the car could take over the control.

This study aims to test the feasibility of a cECG application on the monitoring of patients with artificial cardiac pacemakers and the detectability of pacing events. 20 patients were recruited during their routine visits to the outpatient pacemaker department. The multichannel cECG data collected from these patients wearing their everyday clothing were analyzed using a pacemaker spike detection algorithm.

## 2. Materials and Methods

### 2.1. ECG Recording

**Figure 1:**
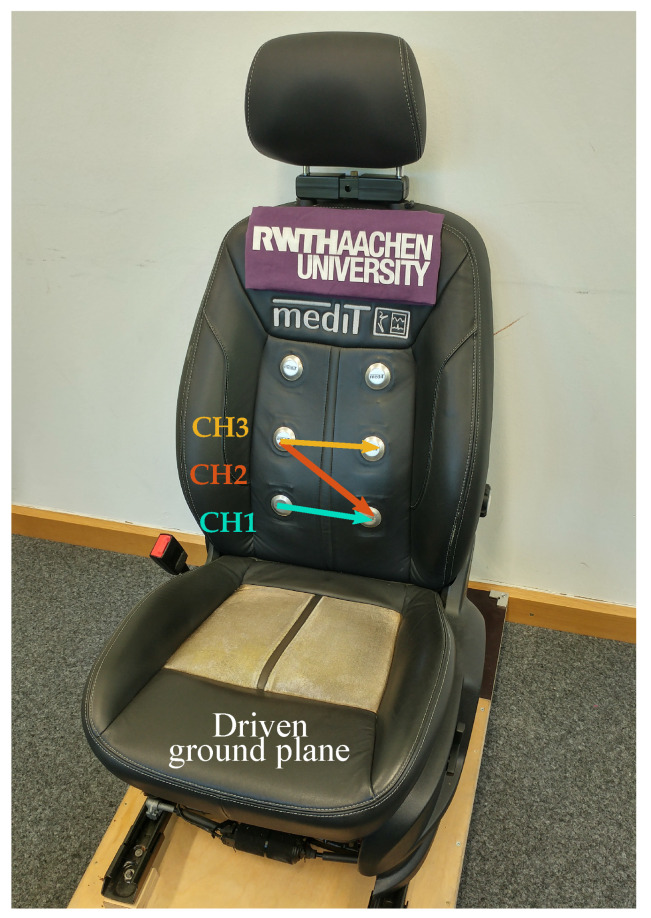
Positions of the capacitive electrocardiogram (ECG) electrodes on the car seat. In the sitting place, the sewn conductive textile was used as a driven ground plane feeding the common-mode signal back to the subject’s body.

**Figure 2:**
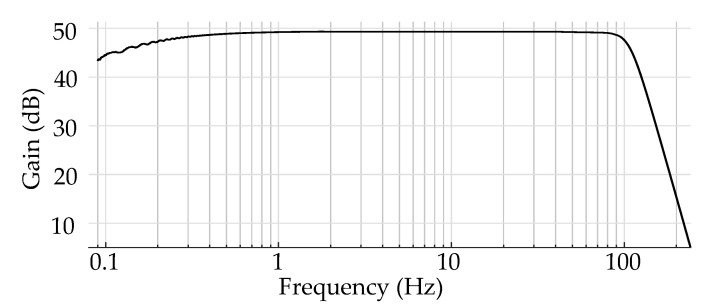
The transfer function of the instrumentation with a gain of 49 dB. The cutoff frequencies were approximately at 0.13 and 102 Hz for 2nd order high-pass and 6th order low-pass Butterworth filters analog domain, respectively.

The cECG system used in this study was based on the car seat presented by Leicht et al. [5] with six cone-formed stainless-steel electrodes, as shown in Figure 1. Each of these electrodes was buffered to make the small amplitude biopotential less susceptible to inferences [6]. Subsequently, different combinations of the electrodes were driven to the instrumentation amplifiers with identical analog channels, according to conventional ECG principles. In addition to stainless-steel electrodes in the backrest, an electrode of silver-coated textile (Statex Produktions- und Vertriebs GmbH, Bremen, Germany) operated as a capacitive driven ground plane electrode at the seating surface. The potential of this electrode was actively driven to the sum of the potential of other electrodes, as in driven right leg electrode in classical ECG [15]. The transfer function of the instrumentation used for the analog signal processing can be seen in Figure 2. The passband gain of the measurement system was approximately 49 dB or 284 V/ V. Since the motion artifacts in cECG can be tens of times higher than the expected amplitude of the cECG signal (approximately 1 mV), the passband gain was kept at this level to avoid clipping due to motion artifacts. Amplified signals were recorded with a sampling rate of 1 kHz using an NI USB-6009-DAQ (National Instruments, Austin, TX, USA). This sampling rate was chosen as 1 kHz to ensure there was enough attenuation to prevent any aliasing and it is exactly two times the sampling rate of the reference system, which makes the resampling numerically more stable.

**Figure 3:**
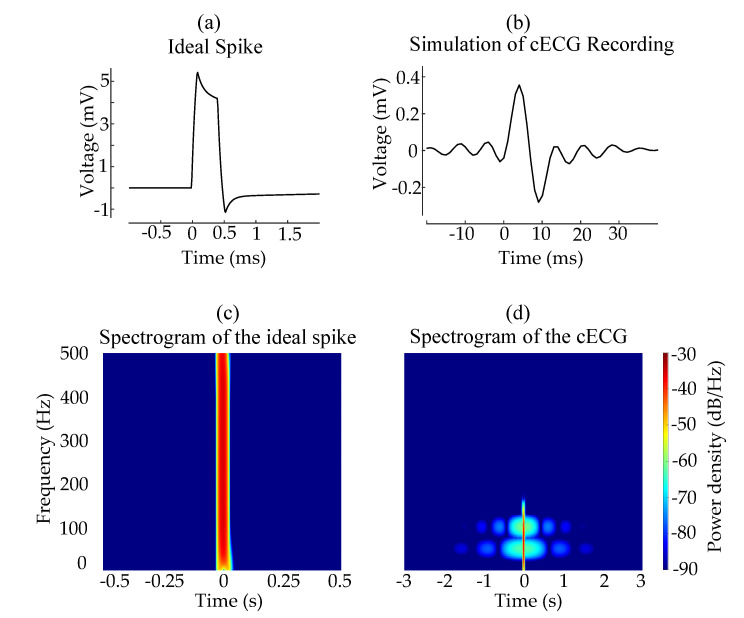
The effect of the analog filters on the spikes of a cardiac pacemaker. The shape of the ideal spike (**a**) is widened with an attenuated amplitude in (**b**), which can be attributed to the low-pass filter in the analog measurement channel. The spectrograms in (**c**,**d**) from the short-time Fourier transform (with Hann window of 64 ms) of the spikes illustrate the impulse-like characteristics of the ideal pacemaker spike and the dampened one of the measurement simulation, respectively. Both spectrograms have the same frequency and color scales but have different time scales to demonstrate the widening better.

After the filters and the amplification in the analog domain as presented in Figure 2, recorded signals were band-pass filtered (0.7 to 140 Hz) in the digital domain and the power-line interference was removed using two band-stop filters at 50 and 100 Hz. An ideal spike shape from a high-resolution ECG [16] was passed through the same processing to emulate the effect of the signal processing on pacemaker spikes. Starting from the analog filters of the system to the zero-phase digital filters used before the evaluation, all processing steps were implemented without adding any measurement noise or power-line interference. The ideal spike with a pulse width of 0.5 ms in Figure 3a was significantly dampened and widened in Figure 3b exhibiting the result of the simulation on the ideal spike shape. From these spikes, two spectrograms were generated using Hann window of 64 ms. The ideal spike shape exhibited an impulse-like spectral density with an almost equally distributed energy over the frequency spectrum at a brief time instance in Figure 3c. On the other hand, the processed spike had a limited frequency spectrum due to the low-pass filter in Figure 3d. Besides, the spike was widened in the time axis due to the band-pass filter and there were ripples caused especially by the band-stop filters. The total decrease in the energy and the dispersion of the energy over a wider time range explain the difference in the amplitudes of the spikes in Figure 3a,b. Zero-phase digital filters caused these ripples in the backward direction too, although they preserved the spike waveform better than causal filters.

A conventional ECG was recorded parallel to the cECG measurement, using a medical-grade bedside patient monitor (Philips MX700, Amsterdam, The Netherlands). This reference ECG (rECG) was employed in the configuration Einthoven Lead-I and has already been digitally processed by the internal proprietary software of the patient monitor. When a pacemaker spike is detected, this digital processing unit overrides the waveform and replaces the section with an ideal shape of a spike. The rECG system was used during the study as the gold standard additionally for its automatic annotation. The recording of both systems was realized separately but simultaneously. By programming the interfaces, both recordings were initialized at the same time, which ensures the time delay between the recordings was always significantly shorter than a single heartbeat. Subsequently, the rECG signal was resampled to 1 kHz and manually aligned with the cECG system to remove possible time delays in the range of few hundreds of ms. The aligned signals were checked at different points to ensure there was no misalignment.

### 2.2. Measurement Protocol

The inclusion criteria of the study were an existing implanted cardiac pacemaker and being older than 65 years. Patients were recruited during their regular follow-up visit to the ambulatory pacemaker clinic at the RWTH Aachen University Hospital (ethical approval under the reference code EK 279/19, ClinicalTrials.gov/NCT04163965).

No special or predefined clothing or textile material was used in order to imitate real-life conditions for measurements. Patients kept their regular clothing, except for coats and thicker outerwear that are unlikely to be worn while driving. They were asked to sit on the cECG seat and three conventional ECG electrodes were attached for the rECG recording. The profile of the study population is given in Table 1.

It should be noted that the clothing of the patients is not grouped according to their materials in Table 1. Dielectric properties of textiles strongly depend on several factors such as frequency, temperature and surface roughness [17]. Note that most sources that present values for the relative permittivity of textiles measured them for the RF-Spectrum (in GHz range) [18] which lies several decades over the ECG spectrum (0.1 Hz to 100 Hz) and which itself spans more than 3 decades. In addition, the human body capacitance and the textile capacitance can be measured by different methods [19,20], each of which has different limitations. An example of potentially misleading permittivity values can be found by comparing the studies in the literature. A range from 1.3 to 1.6 was used as the relative permittivity of cotton for the simulations by Parente et al. [21], whereas the estimated relative permittivity of the same material under the influence of contact pressure yielded values from 2.3 to 4.0 in the measurements of Ueno et al. [22]. Moreover, the manufacturing processes of the same material from the fiber to the yarn or from the yarn to the fabric may lead to different properties, as textiles are very porous [23] and anisotropic materials [24], which alters the mechanical properties of the material as well. Contact pressure on the electrode positions defining the resulting thickness of the clothing depends on the subject’s body composition [25]. Hence, we refrained from grouping patients by clothing without conducting a fully controlled study by providing standardized types of clothing to the subjects, as this would be a misleading simplification of a broader and important discussion. This aspect was kept out of scope, as this study focuses on the feasibility of cECG on patients wearing cardiac pacemakers. Therefore, the clothing of the patients is not grouped. Instead, it is presented the way in Table 1 to show the randomness.

During an examination, the medical staff first check the functionality of the cardiac pacemaker and the occurrence of events since the last examination, by placing the telemetry wand provided by the pacemaker manufacturer over the chest of the patient. This wand enables the communication between the implanted pacemaker and the proprietary software, while the pacemaker is still operating on its own but can be programmed. The staff then continue by testing the sensitivity and capture thresholds to make any necessary changes to the settings of the pacemaker. The amplitude of the pacing stimulus in the performed capture threshold tests is gradually decreased until the pacing does not result in an effective stimulation of the heart, instead inconclusively without atrial or ventricular capture. This point (so-called loss of capture) is used to calculate the necessary amount of stimulus energy so that the stimulus amplitude and duration can be set to a safe level ensuring proper pacing while saving the battery in the meantime. This test can be repeated for different pacing modes of the cardiac pacemaker to test all leads. These threshold tests during the examination of the subjects were marked with the mode of the operation. The rECG and cECG signals were evaluated only during these tests to differentiate different modes of pacing. During the post-processing, short-time artifacts caused by the capacitive coupling between the telemetry wand and the cECG system were observed. As these artifacts were rare and only occurred while programming, they were removed not to cause a false positive spike detection.

The annotations made by the rECG system were grouped into the four modes of pacing utilizing a two-letter nomenclature with the free software SignalPlant [26]. The first letter (U or B) defines the mode of operation as either a uni- or bipolar mode. The second letter (A or V) defines the location of the stimulation as atrial or ventricular as follows:

1. UV: Unipolar ventricular stimulation,
2. BV: Bipolar ventricular stimulation,
3. UA: Unipolar atrial stimulation, or
4. BA: Bipolar atrial stimulation.

Note that this nomenclature, made for the sake of clarity, clusters the rarely occurring left ventricular stimulations in the same way as the right ventricular stimulations, just like the ventricular pacing in any atrioventricular sequential stimulation. This simplification applies to the atrial pacings in the atrioventricular sequential stimulations grouped with right atrial stimulations. Although there were patients with biventricular pacemakers, only isolated pacings of right and left ventricles were recorded during their threshold tests.

### 2.3. Detection Algorithm

The pacemaker spikes in ECGs are distinguishable because of their narrow width and higher amplitude compared to the other elements of a usual ECG waveform. This characteristic shape of the spikes is deteriorated by the processing of the cECG signal, mainly due to the high- and low-pass filters of the analog instrumentation. The low-pass filter limits the bandwidth of the spikes that could otherwise be approximated by an ideal impulse with all possible frequency components. While this widens the spikes, the high-pass filter causes a tail following the spike, which makes spikes look similar to the QRS complex as depicted in Figure 3.

The detection algorithms proposed in the literature were designed for high-resolution ECG with very high sampling rates to overcome this distortion and to benefit from the characteristic shape of the spikes. These detection methods include the fusion of ECG leads and have been tested for different sampling rates of 8 and 32 kHz, the latter of which was found to increase performance [27]. However, as the bandwidth of the ECG broadens, it overlaps with the spectrum of electromyogram (EMG) artifacts. The negative effect of this overlap is found to be lower for higher sampling rates when both clean and EMG-contaminated signals were compared for two distinct sampling rates of 16 and 32 kHz [28]. Moreover, larger bandwidths of the EMG artifacts lead to lower detection rates, as shown in the data with a sampling rate of 10 kHz, which could be overcome by employing non-linear filters enhancing the difference between spikes and EMG artifacts [29].

**Figure 4:**
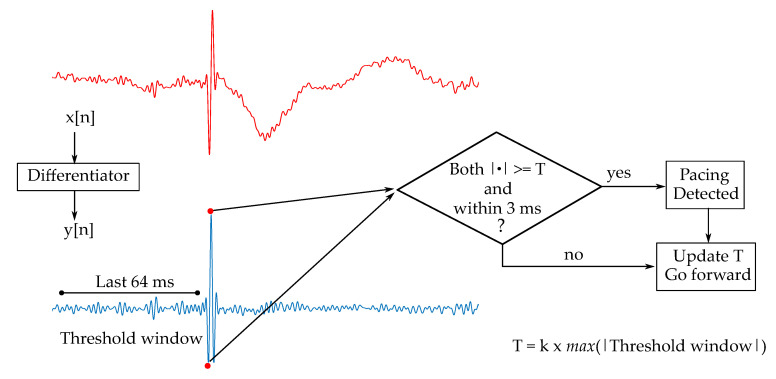
The pacemaker spike detection algorithm proposed by Herleikson [31]. After a differentiator, the algorithm searches for consecutive extrema points with opposite polarities occurring within 3 ms. If the magnitudes of both extrema (marked with red circles) are higher than the adaptive threshold, the instance is classified as a pacemaker spike. The adaptive threshold is calculated as the threshold factor *k* times the highest absolute value in the last 64ms window.

Although the above-mentioned higher sampling rates are beneficial for the pacemaker spike detectability, lower sampling rates such as 500 Hz are more than enough for ECG monitoring. From a practical point of view, lower sampling rates are favored in mobile applications to avoid cumbersome electronics, larger datasets generated, and higher power consumption. A typical cECG application, where the unobtrusiveness is rated higher than the signal quality, would not be equipped with a sampling rate as high as the ones suggested in references [27,28,29]. Therefore, the algorithm used on the system should be suitable for this limited means of diagnostic bandwidth (DBW) ECG. Unlike more sophisticated spike detection algorithms benefiting from larger bandwidths, this study used a pacemaker spike detection algorithm tested on 12-lead ECG limited to DBW with the sampling rate of 500 Hz [30], based on the algorithm patented for patient monitors [31]. This algorithm (Alg_DBW_), illustrated in Figure 4, searches for consecutive edges with opposite polarities and amplitudes *k* times higher than the highest edge in the last 64 ms, after the signal waveform is filtered with the differentiator in Equation (1). The original algorithm, where the consecutive edges must occur within 3 ms, was adapted (Alg_cECG_) to expected cECG spikes with an allowed distance of 4 ms. The threshold factor *k* was swept from 1 to 4.

(1) y[n]=(x[n]+x[n−1])−(x[n−2]−x[n−3]).

### 2.4. Metrics

We introduce a signal quality index (SQI) defined as the average of three quality indices as follows:

(2) $$\begin{array}{cc} \mathrm{basSQI} & =1-\frac{\int_{0\mathrm{Hz}}^{1\mathrm{Hz}}P\left(f\right)df}{\int_{0\mathrm{Hz}}^{40\mathrm{Hz}}P\left(f\right)df}\;, \end{array}$$

(3) $$\begin{array}{cc} \mathrm{qrsSQI} & =\frac{\int_{5\mathrm{Hz}}^{15\mathrm{Hz}}P\left(f\right)df}{\int_{5\mathrm{Hz}}^{40\mathrm{Hz}}P\left(f\right)df}\;, \end{array}$$

(4) $$\begin{array}{cc} \mathrm{pliSQI} & =1-\frac{\int_{49\mathrm{Hz}}^{51\mathrm{Hz}}P\left(f\right)df}{\int_{40\mathrm{Hz}}^{60\mathrm{Hz}}P\left(f\right)df}\;, \end{array}$$

(5) SQI=(basSQI+qrsSQI+pliSQI)/3 ,

where P(f) is the spectral power density.

qrsSQI and basSQI, adapted from Clifford et al. [32], are relative power in the QRS complex and in the baseline wander, respectively. pliSQI is the relative power of the power-line interference. Both pliSQI and basSQI converge to unity due to the 50 Hz band-stop filter and the high-pass filter, except for transients of power-line interference and temporary motion artifacts.

The signal-to-noise ratio (SNR) of the spike is proposed as the ratio of the peak-to-peak amplitude of the pacemaker spike in the cECG ($A_{p-p}$) to the measurement noise ($A_{\mathrm{noise}}$) as follows:

(6) $$\mathrm{SNR}=A_{p-p}/A_{\mathrm{noise}}\;.$$

$A_{p-p}$ depends both on the quality of the electrode contact and the position of the cardiac pacemaker in the thorax regarding the electrodes. $A_{\mathrm{noise}}$ is calculated as the approximate peak-to-peak noise in the close neighborhood of the spike ( 50 ms centered at the spike) and its amplitude increases with increasing contact impedance [33]. Located spikes were set to the value of zero so that only the vicinity of the spike can contribute to $A_{\mathrm{noise}}$. Both of $A_{p-p}$ and $A_{\mathrm{noise}}$ were found using the annotations of the rECG to calculate this metric for each spike in the cECG. This metric is an indicator of how prominent the spike is.

Additionally, true positive rate (TPR) and positive predictive value (PPV) were calculated for the assessment of the detection of cardiac pacemaker spikes:

(7) $$\mathrm{TPR}=\frac{\mathrm{TP}}{\mathrm{TP}+\mathrm{FN}},\;\mathrm{PPV}=\frac{\mathrm{TP}}{\mathrm{TP}+\mathrm{FP}}\;,$$

where a true positive (TP) was defined as a detected spike by the cECG within a tolerance window of 50 ms of an annotated spike by the rECG. Using these definitions, $F_{\beta }$ scores were calculated as

(8) $$F_{\beta }=\left(1+\beta^{2}\right)\;\cdot \;\frac{\mathrm{TPR}\;\cdot \;\mathrm{PPV}}{\mathrm{TPR}+\mathrm{PPV}\;\cdot \;\beta^{2}}\;,$$

where TPR is considered β times as important as PPV. Depending on the application, the operation of the cardiac pacemaker and the underlying pathology of the patient, a false negative detection may be more tolerable than a false positive one or vice versa. For example, in patients with a permanent 3rd degree AV nodal block, 100% ventricular pacing events occur and can be monitored by minimizing false positives. Although there would be more false negatives, the high occurrence rate of pacing events would lead to detected spikes, which could ensure the operation of the pacemaker. On the other hand, a system minimizing false negatives could evaluate the occurrence rate of pacing events of a ventricular-demand pacing mode that operates only when necessary (i.e., inhibition of pacing while detecting an intrinsic heart rhythm in a certain time frame). The occurrence rate of pacings can be estimated from outside while trying to eliminate false positives by comparing the locations of detected spikes with QRS complexes. Due to these possible applications with different focus points, the detection efficiency was evaluated for β values 0.5, 1 and 2, with β being a tuning parameter.

### 2.5. Fusion of the Channels

Unlike classical ECG, the multichannel measurement of cECG does not aim to increase the diagnostic power by capturing different vectors of electrical heart activity. Instead, it strives to increase the time coverage of the cECG measurement by providing redundancy, especially for the case of motion artifacts that are often present in the cECG applications. This redundancy is exploited by different methods in the literature. Injecting a current simultaneously to follow the contact impedances of the channels provides an indicator of the quality of the contact in each channel [34,35]. Another approach is the fusion of these channels at signal-level by using convolutional neural networks [36] or choosing the components of the cardiac activity after blind source separation of the channels [37].

The methods mentioned above require either additional hardware or sophisticated processing of the data, which could deflect the scope and the purpose of this study. Therefore, the channels were evaluated only visually and annotated as *available* or *unavailable* for each segment. This classification was not made to choose the best channel but to eliminate the channels without assessable ECG content. The channels were annotated as *available* as long as the signal was not clipped or the electrode contact was not interrupted.

**Figure 5:**
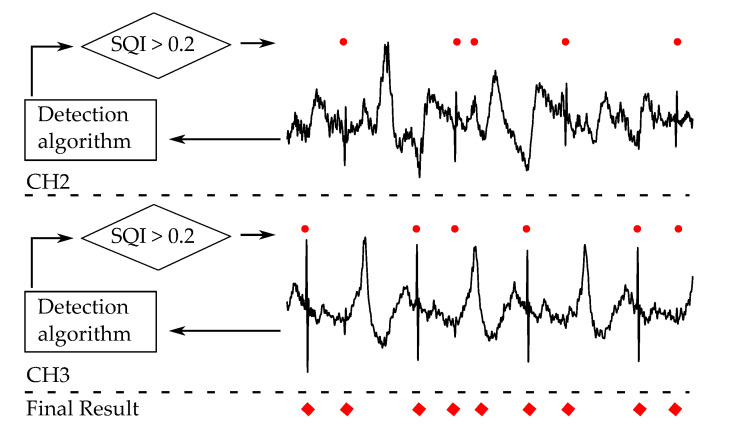
Illustration of the overall fusion algorithm for the detection of the cardiac pacemaker spikes. The resulting spikes (red dots) with an signal quality index (SQI) higher than 0.2 from the channels available (CH2 and CH3) are combined with OR logic and depicted with red diamonds.

The detection algorithm for pacemaker spikes was applied to each available channel. Whenever a spike was detected by the algorithm, SQI was calculated on the time instance of the detection. Detected spikes with an SQI lower than 0.2 were rejected, as a low SQI indicates a low signal quality of the measurement. This threshold value was chosen heuristically such that only the results with significantly lower signal quality were eliminated and the rest stayed untouched. The remaining spikes in different channels were combined with an OR relation. Spikes with less than 20 ms temporal distance were counted as one so that the same spike was not counted twice due to different positions in different channels. The overall scheme of the fusion algorithm is presented in Figure 5.

ECG content assessment of the channels and the fusion scheme were kept simple to keep the focus of the study on the feasibility of the cECG on pacemaker spike detectability. An automated motion artifact detection in cECG channels would influence the results according to its performance, just like a more complex fusion algorithm would do. Both of them are standalone research topics that are not aimed for in this study. Instead, the study is conducted as the test of the cECG measurement technique on a new patient profile.

## 3. Results

**Figure 6:**
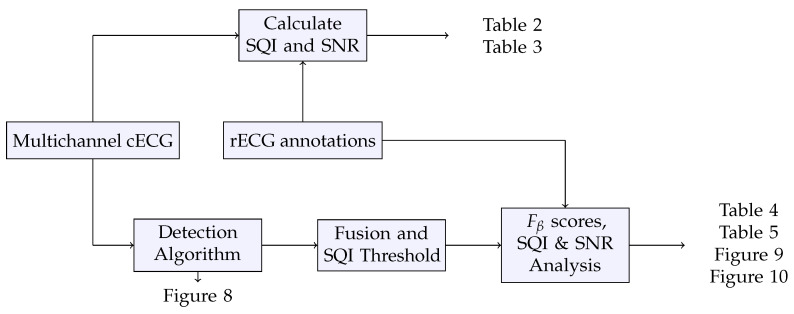
Overview of the workflow generating the results.

The presented results are summarized in Figure 6 depicting the workflow of how each figure and table was generated. Although the particular processes explained the relevant section, this figure was added to provide a clear overview.

### 3.1. Comparison of rECG with cECG

**Figure 7:**
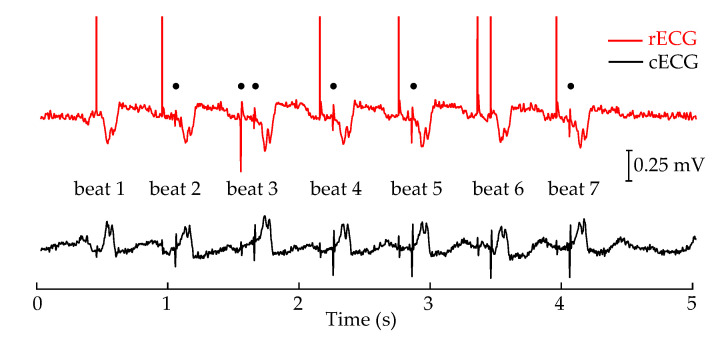
A short section of reference ECG (rECG) and cECG signals serving as an example. When spikes are detected in the rECG by the internal algorithm of the patient monitor, they are irreversibly replaced by an ideal impulse. Undetected spikes (marked with black dots) are present in the beat numbers 2, 3, 4, 5 and 7, which preserved their original shape and amplitude.

A short section of rECG and cECG signals can be seen in Figure 7, extracted from a unipolar atrioventricular sequential stimulation. The spikes in the rECG were digitally processed by the proprietary software of the reference system, which replaced the real spike shapes with an ideal impulse with an amplitude higher than 150 mV. These artificial spikes were clipped in the figure to the amplitude of 0.8 mV for better visualization. Note that the detection algorithm of the reference monitor failed at beats 2–5 and 7, where the unprocessed shapes can be seen in the presented section. Since the mode of the stimulation is unipolar, the spikes exhibit an amplitude of sufficient height to be recognized visually. However, this is not the case for bipolar stimulation. Therefore, only the peaks already detected by the reference system during the threshold tests have been annotated and evaluated in the rest of the results.

An alternative to the annotation by the rECG could be the manual annotations made by a cardiologist but this is not the method chosen. Recordings of the rECG is a single channel electrocardiogram for the visual interpretation. However, its internal software is specifically developed for the detection of pacemaker spikes. Unlike the illustration in Figure 7, the spikes missed by the rECG system are usually not detectable by the human eye, although it can also detect the spikes that are not clearly visible. If the cardiologists had annotated the spikes by visual inspection, no matter how experienced they are, they would have caused a stronger bias in the database of the spikes toward higher amplitudes, as they would not have annotated the ones that are not clearly visible. Therefore, the annotations of the rECG were used but the type and the locations of the annotations were confirmed to be fitting to the changed morphology of the ECG signal by the cardiologists.

### 3.2. Comparison of Signal Quality in cECG Channels

All three quality indices and their mean were calculated for each channel for the segments at which all of them were available to make them comparable. The average value of these indices and their standard deviations are presented in Table 2. These values were not divided among different groups of stimulation, as these metrics relate to the ECG signal quality. CH3 has the highest SQI, although the difference between CH3 and other channels is small. On the other hand, CH1 and CH2 exhibit similar values. The former has a slightly better performance in pliSQI but suffers from the baseline wander more, as can be seen in basSQI. All three channels have similar values in qrsSQI and, thus, the difference in SQI is based mostly upon indices basSQI and pliSQI.

### 3.3. Multichannel Measurement of Cardiac Pacemaker Spikes

**Figure 8:**
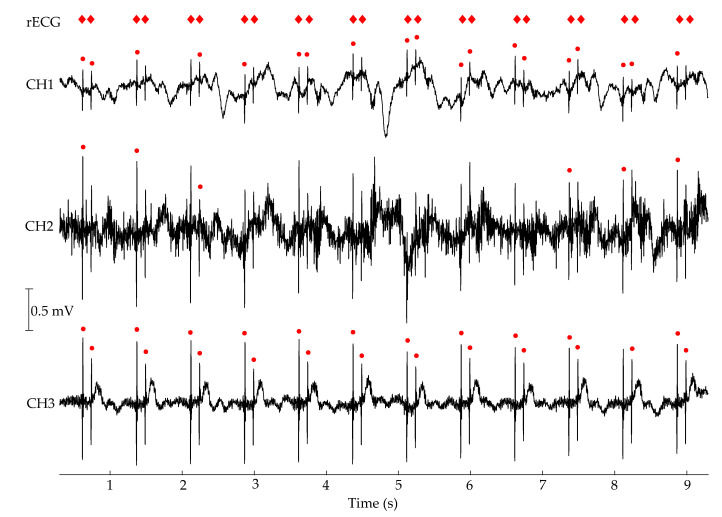
A short section of multichannel cECG measurement during DDD stimulation with three cECG channels. The red circles indicate the results of the spike detection algorithm for each channel, whereas red diamonds are the annotated spikes from the reference system.

A short section of multichannel cECG measurement can be seen in Figure 8, belonging to a segment of unipolar atrioventricular sequential stimulation. Red diamonds on the top indicate the pacemaker spikes annotated by the rECG system. Red dots are the result of the detection algorithm and manifest different rates of success in different channels. CH1 exhibits a more prominent baseline wander, whereas CH2 has a lower SNR.

The profile of the collected cardiac pacemaker spikes by the rECG system is presented in Table 3. The upper part of the table shows the number of cardiac spikes captured in each channel. CH3 has the highest availability among the three channels, covering 3340 from the total 3652 cardiac spikes. CH1 and CH2 cover 48.5% and 54.3% of all spikes, respectively. These availability ratios were calculated for the number of pacings, not for the number of segments. The reason is that segments are not identical and have different lengths, and thus, different numbers of pacings. The lower part of Table 3 presents the average value of the quality metrics related to the cardiac pacemaker spikes. The values were calculated only from the segments at which all channels were available to make the channels comparable. As expected, unipolar stimulations manifest both higher SNR and higher amplitudes than bipolar stimulations. The amplitudes of BA and BV are in the range of the measurement noise in the channels, which gives negative or almost zero SNR values in all channels. CH2 has the highest amplitudes both in UA and UV, although CH3 has higher SNR values. This contradiction is also present in the comparison of CH1 and CH2. Although the spikes in CH2 have higher amplitudes, they have similar SNR values to the spikes in CH1, due to the relatively higher noise in CH2.

### 3.4. Detection Efficiency

**Figure 9:**
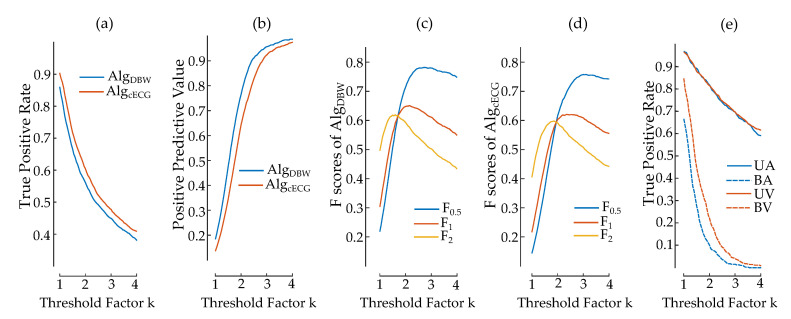
True positive rate (TPR), positive predictive value (PPV) and $F_{\beta }$ scores of the pacemaker spike detection algorithm [31] on cECG measurements. TPR (**a**) and PPV (**b**) are plotted for both Alg_DBW_ (blue) and Alg_cECG_ (orange). Alg_DBW_ (**c**) and Alg_cECG_ (**d**) are evaluated with $F_{\beta }$ scores for three different β values of 0.5, 1 and 2 (blue, orange and gold, respectively). TPR of Alg_cECG_ for different stimulation types (UA, UV, BA and BV) are plotted in (**e**).

The detection efficiency of both Alg_DBW_ and its modified version Alg_cECG_ was tested for different threshold factors *k* from 1 to 4. Figure 9a,b show the TPR and PPV of both algorithms, respectively. Alg_DBW_ exhibits a higher PPV and a lower TPR than Alg_cECG_ at each value of *k*, since Alg_cECG_ accepts wider slopes, which moves the balance between TPR and PPV in the direction of TPR.

The efficiencies of Alg_DBW_ and Alg_cECG_ on cECG data for the three different $F_{\beta }$ scores ($F_{0.5}$, $F_{1}$ and $F_{2}$) are plotted in Figure 9c,d, respectively. Both graphs have a similar shape with different peak points for $F_{0.5}$, $F_{1}$ and $F_{2}$. As the importance shifts from TPR to PPV, that is, from $F_{2}$ to $F_{0.5}$, the locations of the highest scores move to higher *k* values. The highest scores for Alg_DBW_ are at *k* values 1.6, 2.15 and 2.7 for $F_{2}$, $F_{1}$ and $F_{0.5}$, respectively. These points for Alg_cECG_ correspond to *k* values 1.85, 2.35 and 3.1 with the same order. At their respective highest scores, Alg_DBW_ has better scores in $F_{1}$ and $F_{0.5}$, whereas Alg_cECG_ exhibits a similar score in $F_{2}$.

The detection efficiency of different groups is presented in Figure 9e for UA, BA, UV and BV by using Alg_cECG_. Both unipolar stimulations UA and UV (solid lines) have similar detection rates and exhibit a regular decrease with increasing *k* values. On the other hand, the detection rates of bipolar stimulations (dashed lines) BA and BV decrease more rapidly, with BV stimulations having higher detection rates than BA. Unlike TPR, PPV cannot be calculated for different pacing modes, since the detection algorithm used in this study is a binary classifier. Therefore, false positives cannot be assigned to any pacing mode.

Detection scores obtained from the fusion of the channels are presented in Table 4, grouped for different pacing modes at their highest scores. Since false positive spikes cannot be assigned to a specific group, given PPV values are the PPV values of the total collection at the given *k* value. At their optimal settings, Alg_DBW_ is slightly better than Alg_cECG_. By choosing Alg_DBW_ for a cECG application where sensitivity is as important as precision, a *k* value of 2.15 would be the optimal setting using $F_{1}$ score without any *a priori* information of the pacemaker mode. However, it is clear that unipolar and bipolar stimulations have their optimal settings at significantly different *k* values.

### 3.5. Effect of SQI and Fusion on Detection

**Figure 10:**
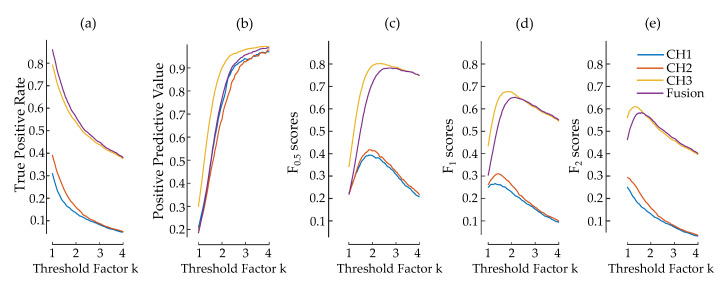
TPR, PPV and $F_{\beta }$ scores of Alg_DBW_ on cECG measurements. TPR (**a**) and PPV (**b**) are plotted for each channel and their fusion separately. $F_{0.5}$, $F_{1}$ and $F_{2}$ are plotted in (**c**,**d**,**e**) respectively.

The effect of the fusion of multichannel cECG was tested by applying Alg_DBW_ on each channel separately. The TPR and PPV for each channel and their fusion are depicted in Figure 10a,b. CH1 and CH2 have significantly lower TPR and PPV values compared to CH3. CH1 is slightly better than CH2 in PPV, whereas CH2 has a moderately higher score in TPR. The fusion of these two channels to CH3 improves TPR slightly and degrades PPV. $F_{\beta }$ scores of the separate channels and the fusion are plotted in Figure 10c–e. CH3 has a better performance than CH1 and CH2 in all $F_{\beta }$ scores and even than the fusion of the channels. The maxima of the $F_{\beta }$ score for each channel are at different *k* values.

After grouping the spikes identified as TP and FN for each channel separately, the difference of the mean of their SQI indices can be seen in Table 5. The number of TPs and FNs in these groups are not paired. Therefore, a two-sample *t*-test with unequal variances was applied to test the significance of the difference of means. The significant results of this test are in bold and marked with an * in the table. These indices, essentially used as quality indices of ECG signals, have a varying indication on the detectability of a pacemaker spike. While pliSQI is significantly higher for TPs, qrsSQI and basSQI are either positive or negative indicators of the detection rate, depending on the channel and the stimulation type.

## 4. Discussion

This proof of concept study shows for the first time that the detection of pacemaker stimulation of the heart with cECG is feasible, with unipolar pacing configuration being superior to bipolar pacing in the detection performance. Multichannel cECG measurements were evaluated by using the annotations provided by the parallel rECG system. However, as shown in Figure 7, the rECG system failed to detect some spikes, which resulted in a limited database. The presented spikes not marked correctly by the rECG system could have been annotated by the cardiologists. However, this post-correction was not possible for most of the missed spikes with lower amplitudes and, therefore, not conducted to ensure comparability. Another side effect of the rECG system is the irreversible processing of the spike shapes, which hinders the comparison between rECG and cECG systems in terms of spike amplitude.

The cECG channels can be compared in two different aspects: The general signal quality and the efficiency in the spike detection. In terms of the ECG quality, CH3 exhibits the least susceptibility to baseline wander and power-line interference, as can be seen from basSQI and pliSQI in Table 2. Moreover, CH3 has the highest coverage in Table 3, supposedly due to its more central position and stable contact with the patients, which provides a balance between contact pressure and the proximity to the heart, as in earlier findings [25]. In terms of spike visibility, CH2 has the highest average amplitude, which can be attributed to its different orientation. However, the higher noise in CH2 makes the spike SNR in CH2 lower than in CH3 in Table 3. Although CH3 appears to be the best among them, it did not have 100% coverage. The gaps in the coverage in a real-world driving scenario could be more dramatic, which makes the fusion of the channels more beneficial by choosing the best channel available.

This study does not present a novel spike detection algorithm. Instead, it aims to show the feasibility of cECG to monitor subjects with cardiac pacemakers and implant activity. Therefore, an algorithm which has already been proposed was used on the cECG signals in two forms: Alg_DBW_ and Alg_cECG_. Alg_cECG_ allows wider spike shapes and exhibits higher detection rates in TPR. However, the original form of the algorithm, Alg_DBW_, leads to higher PPV, due to the more strictly accepted spike width. The same trade-off between TPR and PPV is also present in the choice of the threshold factor *k* in the algorithm. The importance of either one of these two can be different depending on the application of the cECG. Alg_DBW_ is a better alternative for the applications where PPV is favored, whereas Alg_cECG_ captures more spikes at the cost of FPs, as can be seen in Figure 9a,b. The difference between the algorithms in $F_{\beta }$ scores at their respective highest points is insignificant compared to how much *k* values can influence $F_{\beta }$ scores. Therefore, the right adjustment of a *k* value would have a higher impact on the detection efficiency than the adjustment of the spike width within the same algorithm, as in Figure 9c,d.

As the spike amplitude decreases, the threshold factor *k* plays a more crucial role in the detection efficiency, as can be seen in the comparison of bipolar and unipolar stimulations in Figure 9e. The choice of the parameter *k* in this algorithm or any trade-off between TPR and PPV in another algorithm should be made considering the target group of stimulations. Using connected personal healthcare devices, *a priori* information about the expected pacing (bipolar or unipolar) could be used to adapt the algorithm. The benefit of targeting the correct pacing mode is present in Table 4. Without any *a priori* information, Alg_DBW_ optimized for $F_{1}$ score would deliver TPR and PPV values of 0.54 and 0.83, respectively. By separating the pacing modes and using different *k* values, TPR for UA and UV can be increased to 0.71 and 0.72, respectively, while improving their PPV to 0.91. Whereas these scores indicate a competent performance of the cECG system in the pacemaker spike detectability, the scores in bipolar pacing modes exhibit significantly lower values. Therefore, possible improvements in the hardware design, channel fusion, and algorithm development for mobile applications become more crucial for the systems aiming to cover all pacing modes with comparable performance.

CH3 with the highest SQI in Table 2 also has the widest coverage in the number of spikes and the highest SNR in Table 3. However, a direct link between the ECG signal quality and the detection rate is more complex since the comparison of quality indices in Table 5 underlines the different requirements in ECG signal analysis and the detection of pacemaker spikes. Only by evaluating the significant results in Table 5, a higher pliSQI accords with a higher chance of detection. On the other hand, a higher qrsSQI indicates a lower chance of detection, except for bipolar stimulations. This contradictory result can be explained by the definition of qrsSQI, which is the concentration of the energy around the band of the QRS complex. When there is an impulse-like pacemaker spike, the energy is distributed over the spectrum more equally, which reduces qrsSQI. This energy spread effect is more obvious in unipolar stimulations that have higher spike amplitudes, whereas qrsSQI acts as a general quality index in BV stimulations that do not have similarly dominant amplitudes.

The indication of SQIs on the detectability of a spike is variant from channel to channel in Table 5. A higher basSQI is a positive indicator for the detection rates in CH2 and CH3, whereas it correlates negatively in CH1. CH1 is already the channel with the lowest basSQI in Table 2 and has the lowest coverage. An increase in the baseline wander created by the respiratory activity degrades the signal quality in cECG. However, it can be seen as a sign of the coupling strength if the coupling is inherently more susceptible to the respiratory artifacts compared to the coupling in CH2 and CH3, which indicates a higher chance of detection. However, this dichotomy of SQIs in the spike detection and the ECG signal quality should not be directly generalized and needs to be addressed in a controlled experiment, since this study consists of only 20 patients with different physical attributes and pacemaker types.

The fusion of the cECG channels improved TPR slightly and degraded PPV. Therefore, the fusion score is similar to the score of the best channel. However, the fusion scheme in Figure 5 is still beneficial in the automatic choice of the best channel. The fusion of channels was not optimized further as this study focuses on the feasibility of cECG. A decision-level fusion of channels, as in this study, needs to be designed more carefully by taking the different sensitivity of the channels into consideration, since the cECG channels may need to be tuned differently. Instead of fusing all channels, choosing the most suitable channel could also be effective in cECG applications considering that the benefit in TPR does not justify the loss in PPV in the current form of fusion. The more important aspect of the signal fusion in cECG should be identified not as the increase in the diagnostic power but as the increase in the signal availability and time coverage. Therefore, signal-level fusion schemes implementing neural networks [36] and blind source separation methods [38] could be more beneficial.

Another discussion point relates to the properties of the cECG recordings collected in this study. The elderly subjects were recruited and measured in a hospital clinic wearing their everyday clothing. The susceptibility of the measurements to the motion artifacts can be considered lower than a measurement while driving. Due to the wide range of clothing, a generalization in the noise characteristics of the measurements is hardly possible. Power-line interference was removed from the measurements using a digital band-stop filter. However, there are some occasions of transient remnants of this artifact, due to several medical devices operating in the clinic. Consequently, measurements in an automobile environment would have less interference in this frequency range and may achieve better results, as pliSQI is a defining factor in spike SNR and detectability. As required by the study protocol, once the measurement had started, the medical examinations were not interrupted in the case of motion artifacts or any worsening in the electrode contact, due to the anamnesis or the changes in the sitting position of the patients. Therefore, the measurements can be considered a realistic simulation of a cECG system used in ambulatory clinics. Last but not least, the recorded pacemaker spikes belong to the threshold tests. Therefore, their amplitudes decrease gradually by starting from an already low level until the loss of capture, where the pacing amplitude is not enough to result in a cardiac depolarization. In other words, the vast majority of spikes evaluated in this study are weaker than during the normal operation of pacemakers. Hence, higher detectability rates and better signal quality can be expected in the day-to-day operation.

## 5. Conclusions

In this clinical study, spikes of an artificial cardiac pacemaker were recorded using cECG electrodes requiring no direct skin contact. To the best of our knowledge, this is the first application of cECG to humans wearing a cardiac pacemaker. Using the spikes collected from threshold tests, where the pacing amplitude is weaker than normal operation, this study demonstrates the ability of cECG to capture pacemaker spikes and the applicability of this technique in the monitoring of patients with pacemakers. Although the cECG system detected the pacemaker spikes less efficiently than the reference ECG system, it was found to be able to capture the spikes by using an adapted algorithm and a simple fusion strategy. The measurement setup used in this study can easily be extended to other unobtrusive health monitoring applications of cECG, such as chairs and hospital beds.

The analyses revealed the usual signal quality indices used for the signal quality assessment in classical ECG are not necessarily a good indicator of the spike detectability in cECG signals. A more defining factor is the shape of the spike, which is distorted in the analog instrumentation designed for mobile applications with a narrower passband. Both low- and high-pass filters diminish the total energy of the spike and cause phase distortion, which results in wider and dampened spikes. The design considerations of cECG applications, especially in terms of the low-pass filter in the analog signal processing, should be loosened to make cECG more than a QRS detector and a heart rate measurement unit.

In terms of $F_{\beta }$ scores, a wide range of results was obtained, showing the necessity of the careful tuning of algorithm parameters. The different channels of cECG require different settings for their optimal performance between false negatives and false positives, which makes the fusion of these channels essential. The simple decision-level fusion implemented in this study improved the results only in coverage, indicating that a fusion scheme needs to be designed more elaborately or directly in the signal or feature-level. One limitation of this study, as it was kept out of the scope, is dealing with the multichannel measurements in automated decision-making, which needs to be addressed in future work to fully realize a feasible cECG monitoring.

The limitations on the spike detectability using cECG come from the physical limitations and design choices. Its usually higher electrical noise and motion artifacts are physical limitations. On the other hand, the choice of analog filters has a significant effect on the spike amplitude and the intactness of the shape. These translate into our conclusion that unipolar stimulations are easier to detect due to higher amplitudes, comparing to bipolar stimulations. Moreover, the analyses made on the algorithm parameters point to different optimal settings for different types of stimulations, which indicates the necessity of personal healthcare devices with *a priori* information to fully utilize the capabilities of cECG for spike detectability.

## 6. Ethics Statement

All subjects gave their informed consent for inclusion before they participated in the study. The study was conducted in accordance with the Declaration of Helsinki, and the protocol was approved by the Ethics Committee of University Hospital RWTH Aachen (EK 279/19). Experimental procedures involving human subjects in this study were registered at ClinicalTrials.gov with the identifier NCT04163965.

## Figures and Tables

**Table 1:** Patient profile recruited in the study.

Patient	Age	Sex	Height (cm)	Weight (kg)	Pacemaker	Clothing
P1	85	M	176	75	DDD	Cotton undershirt
P2	82	M	182	92	DDD	Cotton undershirt and cotton shirt
P3	84	F	163	85	DDD	Thin blouse of a cotton-polyester mixture
P4	63	F	180	89	DDD	Cotton undershirt and cotton t-shirt
P5	78	M	174	82	DDD	Two layers of cotton undershirt
P6	82	F	158	86	DDD	T-shirt of a cotton-polyester-elastane mixture
P7	80	M	188	95	DDD	Cotton undershirt and cotton t-shirt
P8	73	F	156	55	Bi-vent	Thin blouse of a polyester-cotton mixture
P9	86	F	168	75	DDD	Thin cotton pullover
P10	84	M	171	75	DDD	Cotton undershirt
P11	88	F	177	75	Bi-vent	Cotton undershirt and thin pullover
						of a cotton-polyester mixture
P12	62	F	161	68	DDD	Thick cotton pullover and two layers of
						cotton undershirt
P13	83	M	182	85	DDD	Hospital gown and bathrobe
P14	86	F	154	95	DDD	Pullover of a cotton-polyester mixture
P15	73	M	190	103	DDD	Cotton undershirt
P16	83	F	168	68	DDD	Cotton undershirt and cotton t-shirt
P17	72	F	162	80	DDD	Cotton undershirt and pullover of
						a cotton-polyester mixture
P18	80	M	171	63	DDD	Cotton undershirt and cotton shirt
P19	75	F	164	69	Bi-vent	Cotton undershirt
P20	85	M	158	53	DDD	Cotton undershirt and cotton shirt

**Table 2:** Signal quality indices of cECG channels.

	basSQI	qrsSQI	pliSQI	SQI
CH1	0.25 ± 0.22	0.87 ± 0.11	0.67 ± 0.23	0.60 ± 0.11
CH2	0.36 ± 0.23	0.81 ± 0.17	0.62 ± 0.24	0.60 ± 0.12
CH3	0.52 ± 0.29	0.78 ± 0.17	0.82 ± 0.18	0.71 ± 0.12

**Table 3:** Collection of cardiac pacemaker events annotated by the rECG system.

# of Spikes								
	UA	UV	BA	BV	Total			
CH1	398	691	219	464	1772 (48.5%)			
CH2	427	766	260	529	1982 (54.3%)			
CH3	799	1308	426	807	3340 (91.5%)			
	**SNR (dB)**				**A_p-p_ ( μV)**			
	UA	UV	BA	BV	UA	UV	BA	BV
CH1	5.7	4.5	−1.8	−2.3	100	90	29	25
CH2	4.6	6.7	−1.6	−1.6	155	149	42	43
CH3	13.5	12.0	−1.5	0.6	140	123	11	15

**Table 4:** Optimal settings and detection scores of the stimulation modes for different $F_{\beta }$ scores.

		Optimized for $F_{0.5}$				Optimized for $F_{1}$				Optimized for $F_{2}$			
		*k*	TPR	PPV	$F_{0.5}$	*k*	TPR	PPV	$F_{1}$	*k*	TPR	PPV	$F_{2}$
AlgDBW	BA	1.4	0.23	0.41	0.35	1.3	0.31	0.34	0.32	1	0.56	0.18	0.40
	BV	1.7	0.24	0.61	0.47	1.4	0.41	0.41	0.41	1.1	0.68	0.23	0.49
	UA	3	0.66	0.96	0.88	2.5	0.71	0.91	0.80	1.9	0.80	0.72	0.78
	UV	3	0.67	0.96	0.88	2.45	0.72	0.91	0.80	2	0.78	0.77	0.78
	Total	2.7	0.47	0.93	0.78	2.15	0.54	0.83	0.65	1.6	0.64	0.55	0.62
AlgcECG	BA	1.7	0.17	0.47	0.35	1.4	0.34	0.30	0.32	1.15	0.56	0.19	0.40
	BV	1.85	0.29	0.56	0.47	1.6	0.39	0.41	0.40	1.25	0.67	0.23	0.48
	UA	3.45	0.65	0.95	0.87	2.9	0.70	0.91	0.79	2	0.82	0.65	0.78
	UV	3.35	0.65	0.95	0.87	2.75	0.71	0.89	0.80	2.1	0.79	0.69	0.78
	Total	3.1	0.47	0.93	0.78	2.35	0.54	0.78	0.64	1.85	0.64	0.56	0.62

**Table 5:** Differences of mean SQI of the groups of spikes identified true positive (TP) and FN, where * indicates a difference found significant with α=0.05 in two-sample *t*-test with unequal variances.

		basSQI	qrsSQI	pliSQI	SQI
BA	CH1	**−0.07** *	−0.01	0.05	−0.01
	CH2	0.04	0.00	0.00	0.01
	CH3	**0.17** *	0.02	0.04	**0.08** *
BV	CH1	−0.04	0.01	**0.06** *	0.01
	CH2	−0.03	**0.03** *	**0.06** *	0.02
	CH3	0.03	**0.05** *	0.02	**0.04** *
UA	CH1	**−0.07** *	**−0.03** *	**0.09** *	0.00
	CH2	**0.07** *	**−0.22** *	**0.18** *	0.01
	CH3	**0.17** *	**−0.10** *	**0.13** *	**0.07** *
UV	CH1	**−0.05** *	**−0.03** *	**0.06** *	−0.01
	CH2	0.03	**−0.10** *	**0.10** *	0.01
	CH3	**0.05** *	**−0.07** *	**0.14** *	**0.04** *
